# Molecular Mechanism for Various Pharmacological Activities of NSAIDS

**DOI:** 10.3390/ph3051614

**Published:** 2010-05-25

**Authors:** Tohru Mizushima

**Affiliations:** Graduate School of Medical and Pharmaceutical Sciences, Kumamoto University, 5-1 Oe-honmachi, Kumamoto 862-0973, Japan; E-Mail: mizu@gpo.kumamoto-u.ac.jp; Tel.: +8-196-371-4323; Fax: +8-196-371-4323

**Keywords:** NSAIDs, gastric ulcer, gene expression, cancer, Alzheimer’s disease

## Abstract

The anti-inflammatory action of non-steroidal anti-inflammatory drugs (NSAIDs) is mediated through their inhibitory effects on cyclooxygenase (COX) activity. On the other hand, NSAID use is often associated with gastrointestinal complications. The inhibition of COX by NSAIDs is not the sole explanation for the gastrointestinal side effects of NSAIDs. Furthermore, recent epidemiological studies have revealed that prolonged NSAID use reduces the risk of cancer and Alzheimer’s disease (AD) and a COX-independent unknown mechanism is suggested to be involved in these activities of NSAIDs. In this article, I review our recent work on the COX-independent mechanism involved in NSAID-induced gastric lesions and anti-tumor and anti-AD activities of NSAIDs. Using DNA microarray analysis, we found that NSAIDs affect expression of various genes in a COX-independent manner. We found that membrane permeabilization activity of NSAIDs and resulting NSAID-induced apoptosis are involved in NSAID-induced gastric lesions. On the other hand, induction of expression of tight junction-related genes and endoplasmic reticulum chaperones were suggested to be involved in anti-tumor and anti-AD, respectively, activities of NSAIDs. These results suggest that NSAIDs affect expression of various genes in a COX-independent manner, which is involved in various pharmacological activities of NSAIDs.

## 1. Introduction

Non-steroidal anti-inflammatory drugs (NSAIDs) are one of the most frequently used classes of medicines in the world, accounting for nearly 5% of all prescribed medications [1]. An inhibitory effect of NSAIDs on cyclooxygenase (COX) activity is responsible for their anti-inflammatory actions because COX is an enzyme essential for the synthesis of prostaglandins (PGs), such as PGE_2_, which have a strong capacity to induce inflammation. However, NSAID administration is associated with gastrointestinal complications, such as gastric ulcers and bleeding, which sometimes become life-threatening diseases [2]. About 15-30% of chronic users of NSAIDs have gastrointestinal ulcers and bleeding. In the United States, about 16,500 people per year die as a result of NSAID-associated gastrointestinal complications [3]. Therefore, the molecular mechanism governing NSAID-induced gastrointestinal damage needs to be elucidated in order to develop new NSAIDs that do not have these side effects. 

Inhibition of COX by NSAIDs was previously thought to be fully responsible for their gastrointestinal side effects [4], because PGE_2_ has a strong protective effect on gastrointestinal mucosa [4,5,6,7]. There are at least two subtypes of COX, COX-1 and COX-2, which are responsible for the majority of COX activity at the gastric mucosa and tissues with inflammation, respectively [8,9]. Therefore, it is reasonable to speculate that selective COX-2 inhibitors have anti-inflammatory activity without gastrointestinal side effects. In fact, a greatly reduced incidence of gastroduodenal lesions was reported for selective COX-2 inhibitors (such as celecoxib and rofecoxib) both in animal and clinical data [10]. However, the increased incidence of gastrointestinal lesions and the decrease in PG levels induced by NSAIDs are not always linked with each other. For example, higher doses of NSAIDs were required for producing gastric lesions than were required for inhibiting COX at the gastric mucosa [11]. Understanding the additional mechanisms is necessary in order to establish an alternative method for development of gastrointestinally safe NSAIDs other than simply increasing their COX-2 selectivity. This new class of NSAIDs may be clinically beneficial because clinical disadvantages (ie. risk of cardiovascular thrombotic disease) of selective COX-2 inhibitors were recently suggested [12,13]. 

Recent epidemiologic studies clearly show that NSAID use is associated with a reduced risk of cancer, and preclinical and clinical studies have shown that some NSAIDs are effective for the treatment and prevention of cancer. This effect is particularly well documented in relation to colon and rectal cancer. Recent studies have also shown that NSAID use reduces the risk of stomach cancer [14,15]. A number of different effects of NSAIDs on cancer cells, such as stimulation of apoptosis, cell growth suppression, inhibition of angiogenesis and inhibition of metastasis have been proposed to play important roles in NSAID-mediated chemoprevention [16,17]. However, the precise molecular mechanisms governing these effects of NSAIDs have not been elucidated. 

PGs, such as PGE_2_, inhibit apoptosis and stimulate cell growth, angiogenesis and metastasis [4,18,19]. Furthermore, overexpression of COX-2 has been reported in various tumor cells and tissues [20,21]. Therefore, the inhibition of COX by NSAIDs was previously thought to be the sole explanation for their chemopreventive effect. However, several lines of evidence suggest that chemoprevention by NSAIDs also involves COX-independent mechanisms. Sulindac sulfone, a derivative of the NSAID sulindac, does not inhibit COX activity and has been shown to display anti-tumor activity *in vivo* as well as induce apoptosis and inhibit cell growth in tumor cells *in vitro* [22,23]. Moreover, the induction by NSAIDs of apoptosis and the inhibition of cell growth in COX-null fibroblasts and tumor cells in which COX expression was absent have been reported [24,25]. Therefore, it is important that the COX-independent mechanisms for anti-tumor activity of NSAIDs are elucidated in order to develop more effective NSAIDs. 

Alzheimer’s disease (AD) is pathologically characterized by the accumulation of tangles and senile plaques. Senile plaques are composed of amyloid-β peptides (Aβ) [26,27]. Aβ is generated by secretase-dependent proteolysis of the β-amyloid precursor protein (APP). In order to generate Aβ40 and Aβ42, APP is first cleaved by β-secretase and then by γ-secretase. 

Epidemiological studies have revealed that prolonged use of NSAIDs delays the onset and reduces the risk of AD [28]. In an animal model of AD, administration of some NSAIDs decreased the amount of Aβ and senile plaques and suppressed microglial activation [29,30,31]. Furthermore, in cultured cells, treatment with NSAIDs decreased the amount of Aβ [32,33]. On the other hand, elevated levels of PGE_2_ and overexpression of COX-2 have been observed in the brains of AD patients [34,35,36]. It has also been reported that the extent of COX-2 expression correlates with the amount of Aβ and the degree of progression of AD pathogenesis [37]. Furthermore, transgenic mice that constitutively overexpress COX-2 have been reported to show stimulation of aging-dependent neural apoptosis and memory dysfunction [38]. These results suggest that COX-2 and PGE_2_ are important in the pathogenesis of AD and NSAIDs achieve its anti-AD effect through inhibition of COX. However, the precise molecular mechanisms governing anti-AD effect NSAIDs have not been clear. 

In this study, I review our recent work on COX-independent actions of NSAIDs in order to understand the molecular mechanism for various pharmacological activities of NSAIDs. Results suggest that NSAIDs affect expression of various genes in a COX-independent manner, which is involved in various pharmaceutical activities of NSAIDs. These studies also provide useful information about development of new types of NSAIDs with lower gastrointestinal side effects or with higher anti-cancer (or anti-AD) activity. 

## 2. Results and Discussion

### 2.1. Molecular mechanisms for NSAID-induced gastric lesions

#### 2.1.1. Direct cytotoxic effect of NSAIDs

A number of previous reports suggested that NSAIDs are cytotoxic. Thus, using the primary culture of guinea pig gastric mucosal cells that well mimic the gastric mucosal cells *in vivo* [39], we examined effect of NSAIDs on cell death. We found that short-term or long-term treatments of gastric mucosal cells with NSAIDs induce necrosis or apoptosis, respectively [40,41]. In order to test whether the cytotoxic effect of NSAIDs (necrosis and apoptosis) is dependent of their ability to inhibit COX, we examined the effect of exogenously added PGE_2_ on necrosis and apoptosis induced by NSAIDs. Exogenously added PGE_2_ did not affect the extent of NSAID-induced necrosis or apoptosis even at higher concentrations of PGE_2_ than is present endogenously in medium [42]. Results suggest that the cytotoxic effect of NSAIDs (necrosis and apoptosis) is independent of their ability to inhibit COX [42]. The concentrations of indomethacin required for necrosis and apoptosis *in vitro* were 2.5 and 1 mM, respectively. Studies on the oral administration of indomethacin in rats showed that the concentrations of indomethacin in the stomach were around 1-8 mM when the drug caused gastric injury. Thus, the *in vitro* concentrations of NSAIDs used in our study are physiologically significant *in vivo*.

#### 2.1.2. Membrane permeabilization activity of NSAIDs

**Figure 1:**
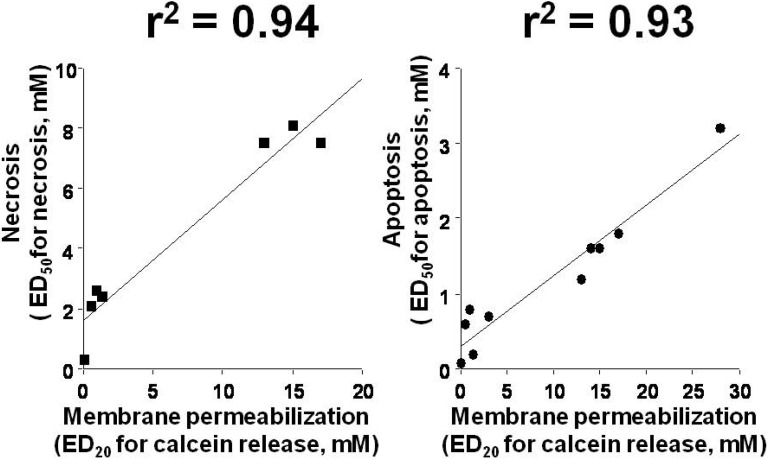
Relationship between necrosis- or apoptosis-inducing and membrane permeabilization activities of NSAIDs. ED_20_ values for membrane permeabilization (calcein release), ED_50_ values for apoptosis and necrosis are calculated and plotted.

Based on the structure of NSAIDs, we hypothesized that NSAIDs have membrane permeabilization activity and this activity is involved in the cytotoxic effect of NSAIDs, in other words, the primary target of NSAIDs is the membrane. We examined membrane permeabilization activity of more than 10 NSAIDs (nimesulide, celecoxib, mefenamic acid, flufenamic acid, flurbiprofen, indomethacin, diclofenac, etodolac, ibuprofen and ketoprofen) using calcein-loaded liposomes [43,44]. Calcein fluoresces very weakly at high concentrations due to self-quenching, so the addition of membrane permeabilizing drugs to a medium containing calcein-loaded liposomes should cause an increase in fluorescence by diluting out the calcein [43]. All of the NSAIDs tested increased the calcein fluorescence, suggesting that they have membrane permeabilization activity [43,44]. We also examined the necrosis- and apoptosis-inducing ability of these NSAIDs. To examine the relationship between NSAID-induced necrosis or apoptosis and membrane permeabilization, we determined ED_50_ values of the 10 NSAIDs for necrosis or apoptosis (concentrations of NSAIDs required for 50% inhibition of cell viability by necrosis or apoptosis) and ED_20_ values for membrane permeabilization (concentration of NSAIDs required for 20% release of calcein). Plotting ED_50_ values for necrosis or apoptosis vs ED_20_ values for membrane permeabilization (calcein release) yielded an r^2^ value of 0.94 or 0.93, respectively (Figure 1), which suggests that NSAID-induced necrosis and apoptosis is mediated by their ability to permeabilize membranes.

#### 2.1.3. DNA microarray analysis

It is believed that necrosis is induced by drastic permeabilization of cytoplasmic membranes, however, the mechanisms underlying the how membrane permeabilization activity of NSAIDs induces apoptosis remained unclear. In order to understand molecular mechanism governing this apoptosis, we searched for genes whose expression is induced by indomethacin using DNA microarray analysis and found that CHOP, a transcription factor with apoptosis-inducing ability is induced by various NSAIDs [45,46,47]. In order to test whether the induction of CHOP by indomethacin is involved in indomethacin-induced apoptosis, we used CHOP deficient mice. Indomethacin-induced chromatin condensation was observed in peritoneal macrophages from wild-type mice but not so apparently in those from CHOP deficient mice [46]. This result strongly suggests that the induction of CHOP is involved in NSAID-induced apoptosis. 

#### 2.1.4. Contribution of the increase in intracellular Ca^2+^ level and mitochondrial dysfunction to NSAID-induced apoptosis

Permeabilization of cytoplasmic membranes causes an increase in intracellular Ca^2+^ levels by stimulating Ca^2+^ influx across the cytoplasmic membrane and we showed that all of NSAIDs tested increase the intracellular Ca^2+^ level [44]. We used BAPTA-AM, an intracellular Ca^2+^ chelator that is permeable for cytoplasmic membranes to test the contribution of the increase to NSAID-induced apoptosis. BAPTA-AM inhibited NSAID-induced cell death, apoptotic chromatin condensation and induction of CHOP [44], suggesting that the increase in intracellular Ca^2+^ levels caused by NSAIDs is involved in NSAID-induced CHOP induction and resulting apoptosis.

CHOP is induced by endoplasmic reticulum (ER) stress response and thus results described above show that NSAIDs induce ER stress response. Accumulation of unfolded protein in the ER induces the ER stress response. On the other hand, PUMA (p53 up-regulated modulator of apoptosis) is a BH3 only domain protein with potent apoptosis-inducing activity [48,49]. PUMA stimulates conformational change, translocation and multimerization of Bax that permeabilizes the mitochondrial outer membrane, resulting in mitochondrial dysfunction and apoptosis [50]. It was recently reported that ER stressors (such as tunicamycin and thapsigargin) up-regulate PUMA [51,52]. Based on these previous reports, we considered that PUMA could link the ER stress response to mitochondrial dysfunction in the NSAID-induced apoptosis pathway. 

We found that various NSAIDs up-regulate PUMA [53]. Small interfering RNA (siRNA) specific for PUMA revealed that PUMA up-regulation is involved in NSAID-induced activation and translocation of Bax, mitochondrial outer membrane permeabilization, and induction of apoptosis [53]. Furthermore, we suggested that NSAID-induced up-regulation of PUMA is mediated through an increase in the intracellular Ca^2+^ level, up-regulation of ATF4 and CHOP [53].

**Figure 2:**
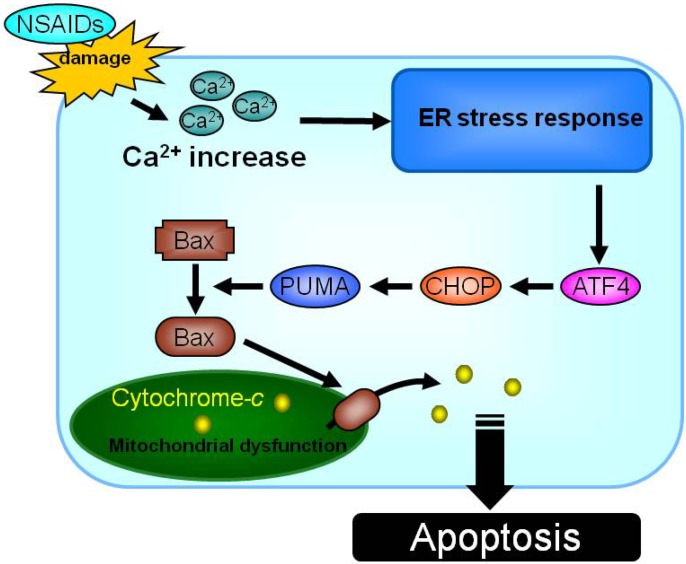
Molecular mechanism for NSAID-induced apoptosis.

Based on these results, we proposed the following pathway for NSAID-induced apoptosis (Figure 2). Permeabilization of cytoplasmic membranes by NSAIDs stimulates Ca^2+^ influx and increases intracellular Ca^2+^ levels, which in turn induces the endoplasmic reticulum (ER) stress response. In the ER stress response, expression of CHOP and ATF4 are induced to induce the expression PUMA and the resulting translocation and activation of Bax. Bax plays an important role in NSAID-induced mitochondrial dysfunction, activation of caspases and apoptosis. 

#### 2.1.5. Contribution of mucosal cell death to NSAID-induced gastric lesions

We considered that not only COX inhibition (inhibition of PG synthesis) but also the COX-independent direct cytotoxic effect of NSAIDs is involved in the development of gastrointestinal lesions *in vivo*. For testing this idea by pharmacological experiments, it is necessary to separate these two properties of NSAIDs (ie. COX inhibition and direct cytotoxicity) in the model of NSAID-induced gastric lesions *in vivo*. We tried to achieve this by employing intravenous administration of a non-selective NSAID (indomethacin) and oral administration of cytotoxic selective COX-2 inhibitors (such as celecoxib) in rats. Intravenous administration of non-selective NSAIDs may cause inhibition of both COX-1 and COX-2 (thus inhibition of PG synthesis) at the gastric mucosa without any direct cytotoxicity to the gastric mucosa, because the concentration of NSAIDs at the gastric mucosa following intravenous administration is much lower compared to when NSAIDs are orally administered. On the other hand, oral administration of celecoxib may cause direct cytotoxicity to the gastric mucosa without inhibition of COX-1 and thus PG synthesis may be maintained. 

**Figure 3:**
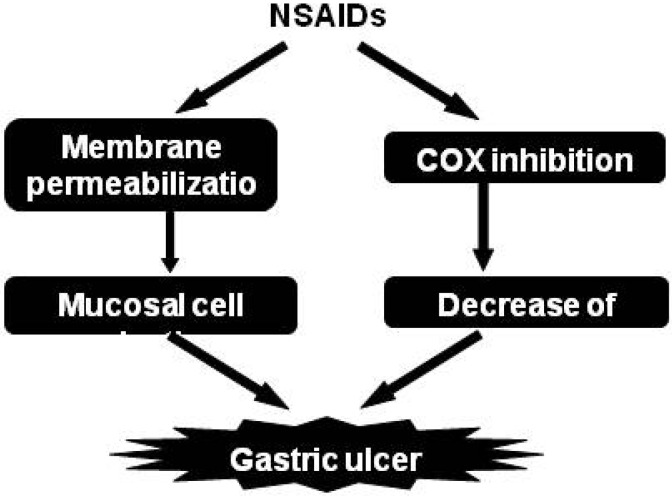
Mechanism for NSAID-induced gastric lesions.

Intravenously administered indomethacin, which completely inhibited COX activity at the gastric mucosa, did not produce gastric lesions. Orally administered celecoxib also did not produce gastric lesions. Interestingly, a combination of the oral administration of celecoxib with the intravenous administration of indomethacin clearly produced gastric lesions [42]. These results suggest that in addition to COX-inhibition by NSAIDs, direct cytotoxicity of NSAIDs is involved in NSAID-induced gastric lesions (Figure 3). 

#### 2.1.6. Strategy for development of NSAIDs with lower gastrointestinal side effect

Results described above suggest that NSAIDs without membrane permeabilizing activity have reduced gastrointestinal side effects. As described above, an issue that was recently raised concerning the use of COX-2-selective NSAIDs is their potential risk for promoting cardiovascular thrombotic events [13]. Prostacyclin, a potent anti-aggregator of platelets and a vasodilator, is mainly produced by COX-2 in vascular endothelial cells, while thromboxane A_2_, a potent aggregator of platelets and a vasoconstrictor, is mainly produced by COX-1 in platelets [54]. Until recently, rofecoxib and celecoxib were leading COX-2 selective NSAIDs in the market. Rofecoxib was withdrawn from the market due to the risk of it promoting cardiovascular thrombotic events and the U. S. Food and Drug Administration (FDA) advised physicians to consider alternatives to celecoxib due to the risk of it causing cardiovascular thrombotic events [55]. Based on our findings, NSAIDs that do not exhibit membrane permeabilization activity may be safe for the gastrointestinal tract even if they are not highly selective for COX-2. This type of NSAID (without membrane permeabilization activity and COX-2 selectivity) may be of clinical benefit because they are predicted to be safe for both the gastrointestinal tract (due to less membrane permeabilization activity) and cardiovascular system (due to less COX-2 selectivity). We are now chemically synthesizing such NSAIDs.

However, since development of a new drug requires very long period, we should consider what to do with existing medicines. One possible way is to use NSAIDs with relatively low direct cytotoxicity. We recently showed that nabumetone and nitric oxide-releasing indomethacin have relatively low direct cytotoxicity [56,57].

The other strategy is to use gastroprotective drugs that prevent NSAID-induced cell death. We have proposed that geranylgeranylacetone (GGA, a leading anti-ulcer drug in Japanese market and a non-toxic heat shock protein (HSP)-inducer [58,59]) is beneficial for preventing NSAID-induced gastric lesions. 

When cells are exposed to stressors, a number of so-called stress proteins are induced, in order to confer protection against such stressors. Heat shock proteins (HSPs) are representative of these stress proteins, and their cellular up-regulation, especially that of HSP70, provides resistance [60]. The up-regulation of HSPs by various stressors is regulated at the transcription level by a transcription factor (heat shock factor 1 (HSF1)) [61]. We have previously reported that pre-induction of HSPs by GGA protects cultured gastric mucosal cells from cell death induced by NSAIDs [62,63]. We recently reported that indomethacin-induced gastric lesions were ameliorated in transgenic mice expressing HSP70 [64]. After indomethacin administration, fewer apoptotic cells were observed in the gastric mucosa of transgenic mice expressing HSP70 than in wild-type mice and suppression of HSP70 expression *in vitro* stimulated indomethacin-induced apoptosis and activation of Bax but not the ER stress response, suggesting that expression of HSP70 confers gastric protection against indomethacin-induced lesions by inhibiting the activation of Bax and resulting apoptosis [64]. GGA induced HSP70 at the gastric mucosa in an HSF1-dependent manner and suppressed the formation of indomethacin-induced gastric lesions in wild-type mice but not in HSF1-null mice, suggesting that the HSP-inducing activity of GGA seems to contribute to its gastro-protective activity [41,64,65]. We also reported that GGA is therapeutically beneficial against inflammatory bowel disease (IBD)-related colitis and lesions of small intestine through its HSP-inducing ability [66,67]. 

### 2.2. Molecular mechanisms for anti-tumor activity of NSAIDs

#### 2.2.1. Role of tight junction-related genes in anti-tumor activity of NSAIDs

As described above, NSAID-induced apoptosis is one of the most important mechanisms for the anti-tumor effect of NSAIDs, therefore, the mechanism for NSAID-induced apoptosis described above should play an important role in anti-tumor effect of NSAIDs.

Tight junctions (TJs) are the most apical intercellular structure in epithelial and endothelial cells and create a physiological barrier separating the apical and basolateral spaces; in other words, they create a paracellular permeability barrier. TJs contain the transmembrane proteins occludin and claudin, which are connected to the cytoskeleton via zonula occludens [68]. A number of studies have demonstrated a correlation between a reduction in TJ function and tumor progression. A loss of TJ structure is frequently observed in epithelium-derived cancers, while some tumor-promoting agents are known to disrupt TJs [69,70]. Furthermore, overexpression of tight junction-related proteins (such as claudin-1, claudin-4 and occludin) in cancer cells has been reported to induce apoptosis and suppress the invasive potential of these cells [71,72].

DNA microarray analysis described above also revealed that genes related to TJ function (claudin-1, claudin-4 and occludin) are induced by indomethacin in human gastric carcinoma (AGS) cells [45]. We focused on claudin-4 because the induction was relatively clear. Induction of claudin-4 by indomethacin was confirmed at both the mRNA and protein levels and NSAIDs other than indomethacin (diclofenac and celecoxib) also induced claudin-4 [45]. To identify the mechanism for induction of claudin-4 by NSAIDs, we tested whether NSAID-induced increase in the intracellular Ca^2+^ concentration is involved in this induction. Other drugs that increase the intracellular Ca^2+^ concentration (thapsigargin and ionomycin) also induced claudin-4 and BAPTA-AM inhibited the indomethacin–dependent induction of claudin-4, strongly suggesting that induction of claudin-4 by indomethacin is mediated through an increase in the intracellular Ca^2+^ concentration [45]. 

The migration activity of tumor cells is very important for tumor progression. Addition of indomethacin or overexpression of claudin-4 inhibited AGS cell migration and suppression of claudin-4 expression by siRNA restored the migration activity of AGS cells in the presence of indomethacin. [45]. The anchorage-independent growth of tumor cells, which can be measured by colony formation in soft agar, is also important for tumor progression. Colony formation in soft agar was inhibited by overexpression of claudin-4 [45]. Based on these results, we consider that the induction of claudin-4 is involved in the anti-tumor effect of NSAIDs thorough the suppression of anchorage-independent growth and cell migration.

Next, we have systematically examined the effects of various NSAIDs on the expression of various tight junction proteins and have found that NSAIDs specifically and drastically inhibit the expression of claudin-2 [73]. Being different from the case of claudin-4, BAPTA-AM did not affect the suppression of expression of claudin-2 by NSAIDs, suggesting that suppression of expression of claudin-2 by NSAIDs is not mediated through an increase in the intracellular Ca^2+^ level [73]. Overexpression or suppression of claudin-2 expression caused stimulation or inhibition, respectively, of the invasion and migration activity of AGS cells [73]. Furthermore, NSAIDs inhibited the invasion and migration activity of cancer cells and this inhibition was suppressed by overexpression of claudin-2 [73]. These results suggest that inhibition of claudin-2 expression by NSAIDs contributes to NSAID-dependent inhibition of invasion of cancer cells *in vitro* and that it may be involved in the anti-tumor effects of NSAIDs by inhibiting metastasis *in vivo*. 

Thus, it seems that depending on the claudin species, claudins positively or negatively affect the progression of cancer through various mechanisms. Screening of NSAIDs to identify molecules that potently induce claudin-4 expression and suppress claudin-2 expression may be useful for obtaining more potent NSAIDs for cancer treatment. Although NSAIDs and TJs are known to be closely associated in relation to cancer progression, this is the first time that a connection between NSAIDs and TJs has been shown at the molecular level. 

#### 2.2.2. Roles of ER chaperones in anti-tumor activity of NSAIDs

In ER stress response, in addition to induction of apoptosis by CHOP, ER chaperones (such as glucose-regulated protein (GRP)-78 and 150-kDa oxygen-regulated protein (ORP150)) are induced to protect the ER against ER stress by refolding unfolded proteins in the ER [74]. It was reported that overexpression of GRP78 makes cells resistant to apoptosis induced by anti-cancer drugs (topoisomerase inhibitors) and ER stressors (tunicamycin and Ca^2+^ ionophores) [75,76]. Furthermore, recent papers have described the up-regulation of ORP150 in clinically isolated tumors and cancer cell lines [77,78]. Therefore, we examined the role of ER chaperones in anti-tunmor effect of NSAIDs by examining the effect of ER chaperones on NSAID-induced apoptosis. At first we showed that NSAIDs, including celecoxib, an NSAID that is particularly effective in the treatment and prevention of cancer [79], up-regulated the expression of GRP78 and ORP150 in cultured AGS cells [47,80]. Celecoxib also up-regulated GRP78 and ORP150 in xenograft tumors, accompanied by the suppression of tumor growth in nude mice [47,80]. Suppression of ATF4 expression by siRNA or BAPTA-AM inhibited the celecoxib-dependent up-regulation of GRP78 and ORP150, suggesting that the Ca^2+^-dependent activation of the ATF4 pathway is involved in the up-regulation of ER chaperones by celecoxib [47,80]. 

Overexpression of GRP78 or ORP150 partially suppressed the apoptosis and induction of CHOP in the presence of celecoxib but not that of staurosporine, which does not induce ER chaperones [47,80]. On the other hand, suppression of GRP78 or OPR150 expression by siRNA drastically stimulated cellular apoptosis and production of CHOP in the presence of celecoxib [47,80]. These results show that up-regulation of ER chaperones by celecoxib protects cancer cells from celecoxib-induced apoptosis, and thus may decrease the potential anti-tumor activity of celecoxib.

#### 2.2.3. Roles of S100P in anti-tumor activity of NSAIDs

DNA microarray analysis described above also revealed that indomethacin induces the expression of S100P. S100P is a member of the S100 family of EF-hand Ca^2+^-binding proteins [81]. Overexpression of S100P has been observed in tumors clinically isolated from various tissue types, with the extent of the overexpression being positively correlated to the degree of malignancy [82,83,84,85,86,87]. Overproduction of S100P appears to stimulate tumor malignancy through both intracellular and extracellular mechanisms [88]. Secreted S100P binds to its receptor, the receptor for activated glycation end products (RAGE), thereby stimulating the invasion and growth of cancer cells or inhibiting their apoptosis through activation of extracellular-regulated kinase (ERK) and nuclear factor-B (NF-B) [84,89,90,91]. Furthermore, S100P was suggested to function also in cells through its binding to ezrin and Casy/SIP [92,93,94]. On the other hand, the mechanism for regulation of expression of S100P was unclear. Thus, we examined the molecular mechanism for NSAID-induced expression of S100P and the role of S100P in anti-tumor effect of NSAIDs. We confirmed the up-regulation of S100P expression in AGS cells treated with various NSAIDs, including celecoxib [95]. The celecoxib-mediated up-regulation of expression of S100P was suppressed by the transfection of cells with siRNA for ATF4 and deletion of ATF4-binding consensus sequence located in the promoter of the *S100P* gene resulted in inhibition of celecoxib-mediated transcriptional activation of the gene, suggesting that celecoxib up-regulates the expression of S100P through an ATF4-mediated ER stress response [95].

Inhibition of the growth and induction of apoptosis by celecoxib was either suppressed or stimulated by transfection of cells with S100P overexpression plasmid or siRNA, respectively [95]. Celecoxib also inhibited the invasive activity of the cells [95]. Cromolyn, which inhibits the binding of S100P to its receptor, enhanced the celecoxib-mediated inhibition of cell invasion and growth, but did not affect apoptosis [95]. These results suggest that S100P affects apoptosis, cell growth and invasion through either an intracellular or an extracellular mechanism, and that the up-regulation of S100P expression by NSAIDs reduces their anti-tumorigenic activity.

#### 2.2.4. Strategy for development of NSAIDs with potent anti-tumor activity

**Figure 4:**
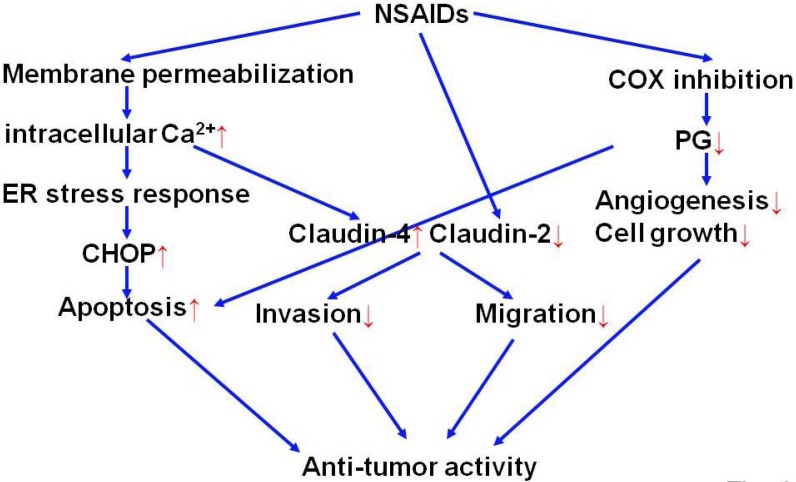
Mechanism for anti-tumor activity of NSAIDs.

The proposed mechanism for anti-tumor activity of NSAIDs is shown in Figure 4. Although we focused on COX-independent mechanisms, it is certain that COX-inhibition by NSAIDs is important for anti-tumor activity of NSAIDs. Thus, NSAIDs with potent inhibitory activity on COX, especially COX-2, frequently overexpressed in tumors, is beneficial. However, risk of cardiovascular thrombotic disease of selective COX-2 inhibitors is of concern [12,13]. 

On the other hand, since induction of expression of claudin-4 and CHOP inhibit the invasion and induce apoptosis, respectively, of cancer cells and the induction is regulated by increase in the intracellular Ca^2+^ concentration, NSAIDs with potent activity to increase the intracellular Ca^2+^ concentration may be beneficial as anti-tumor drugs. However, we also showed that induction of expression of ER chaperones and S100P negates the inhibition of invasion and induction of apoptosis, respectively, of cancer cells and this induction is regulated by increase in the intracellular Ca^2+^ concentration and resulting ER stress response. Since ER stress response is regulated by three types of ER transmembrane proteins: protein-kinase and site-specific endoribonuclease, protein kinase R-like ER kinase and activating transcription factor 6 [96,97,98] and each ER stress response-related gene is regulated differently by these three pathways, identification of exact pathway for induction of expression of each of CHOP, cluadin-4, ER chaperones and S100P is important for establishing the strategy for development of NSAIDs with potent anti-tumor activity. It is also important to identify the pathway for NSAID-dependent suppression of expression of claudin-2.

### 2.3. Molecular mechanisms for anti-AD activity of NSAIDs

#### 2.3.1. Roles of COX-inhibition and PG-decrease in anti-AD activity of NSAIDs

As described in the introduction, anti-AD activity of NSAIDs is believed to mainly involve the COX-inhibition and PG-decrease. In order to obtain direct evidence in support of this concept, we examined the effect of PGE_2_ on production of Aβ *in vitro*. PGE_2_ stimulated the production of Aβ in cells expressing a mutant type of APP [99]. PGE_2_ receptors have been pharmacologically subdivided into four main subtypes (EP_1_, EP_2_, EP_3_ and EP_4_) [100]. We have demonstrated using subtype-specific agonists that EP_2_ and EP_4_ receptors are responsible for this PGE_2_-stimulated production of Aβ [99]. 

Activation of EP_2_ and EP_4_ receptors is coupled to an increase in cellular cAMP levels and activation of PKA. We found that inhibitors of adenylate cyclase and PKA suppress EP_2_ receptor- but not EP_4_ receptor-mediated stimulation of the Aβ production. In contrast, inhibitors of endocytosis suppressed EP_4_ receptor- but not EP_2_ receptor-mediated stimulation [101]. PGE_2_-dependent internalization of the EP_4_ receptor was observed and cells expressing a mutant EP_4_ receptor lacking the internalization activity did not exhibit PGE_2_-stimulated production of Aβ [101]. A physical interaction between the EP_4_ receptor and PS-1, a catalytic subunit of γ-secretases, was revealed by immunoprecipitation assay [101]. PGE_2_-induced internalization of PS-1 and co-localization of EP_4_, PS-1 and Rab7 (a marker of late endosomes and lysosomes) were observed [101]. These results suggest that PGE_2_-stimulated production of Aβ involves EP_4_ receptor-mediated endocytosis of PS-1 and EP_2_ receptor-dependent activation of adenylate cyclase and PKA. 

Transgenic mice expressing the mutant type of APP showed lower levels of A in the brain, when they were crossed with mice lacking either EP_2_ or EP_4_ receptors, suggesting that PGE_2_-mediated activation of EP_2_ and EP_4_ receptors is involved in the production of A *in vivo* and in the pathogenesis of AD [99]. These results suggest that NSAIDs achieve its anti-AD activity through COX-inhibition and PG-decrease.

#### 2.3.2. Role of ER chaperones in anti-AD activity of NSAIDs

APP first translocates into the ER where they undergo modification, which is essential for the generation of Aβ [102]. The ER is also proposed to be important for Aβ-induced apoptosis of neuronal cells [103,104]. These observations suggest that the ER is an important cellular compartment for the pathogenesis of AD. Therefore, it is reasonable to speculate that ER chaperones affect the generation of Aβ and the pathogenesis of AD. In fact, accumulation of GRP78 in senile plaque, up regulation of ER chaperones in brains of AD patients, and co-localization of ER chaperones with Aβ have all been reported [105,106,107]. Thus, we consider that induction of ER chaperones by NSAIDs is involved in the anti-AD activity of NSAIDs and we systematically examined the effect of overexpression of various ER chaperones on the generation of Aβ *in vitro*. Overexpression of GRP78 or inhibition of its basal expression decreased or increased, respectively, the level of Aβ40 and Aβ42 in conditioned medium [108]. In the case of the other ER chaperones, overexpression of some (ORP150 and calnexin) but not others (GRP94 and calreticulin) suppressed the production of Aβ [108]. These results indicate that certain ER chaperones are effective suppressors of Aβproduction. GRP78 was co-immunoprecipitated with APP and overexpression of GRP78 inhibited the maturation of APP, suggesting that GRP78 binds directly to APP and inhibits its maturation, resulting in suppression of the proteolysis of APP [108]. These results suggest that induction of ER chaperones by NSAIDs is involved in the anti-AD activity of NSAIDs.

#### 2.3.3. Strategy for development of NSAIDs with potent anti-AD activity

As described above, NSAIDs have attracted much attention as a new class of drugs for the treatment and prevention of AD. In combination with other studies suggesting that the COX-independent actions of NSAIDs (such as direct binding to γ-secretase, activation of the peroxisome proliferators activated receptor-γPPARγ and resulting inhibition of β-secretase, inhibition of Rho/Rho kinase (Rock) pathway, and activation of nuclear factor-κB (NF-κB)) are involved in the inhibitory effect of NSAIDs on the production of Aβ [30,32,33,109], COX-inhibition and the resulting decrease in the level of PGE_2_, is also involved in the anti-AD activity of NSAIDs. At present, although the importance of COX-inhibition in the anti-AD activity of NSAIDs is clear, the importance of COX-independent mechanism is not so clear. Identification of important mechanism for anti-AD activity of NSAIDs is required for establishing the strategy for development of NSAIDs with potent anti-AD activity.

## 3. Conclusions

Results of our recent studies reviewed in this article suggest that both COX-dependent and COX-independent mechanisms are involved in various pharmacological activities of NSAIDs. I want to also note that examination of the action of existing pharmacological agents (such as NSAIDs) reveals various physiological mechanisms, such as contribution of TJs to migration of cells and contribution of receptor internalization to production of Aβ. 
